# Sleep, executive functions, and repetitive behaviors in autism spectrum disorder: different patterns of association with lower- and higher-level repetitive behaviors

**DOI:** 10.3389/fpsyg.2026.1903369

**Published:** 2026-10-07

**Authors:** Elisa Fucà, Martina Parisi, Silvia Guerrera, Stefano Vicari

**Affiliations:** 1 Child and Adolescent Neuropsychiatry Unit, Bambino Gesù Children's Hospital, IRCCS, Rome, Italy; 2 Life Sciences and Public Health Department, Catholic University, Rome, Italy

**Keywords:** emotional regulation, insistence on sameness, insomnia, motor stereotype, shift

## Abstract

**Background:**

Restricted and repetitive behaviors (RBs) are a core feature of autism spectrum disorder (ASD), although the mechanisms underlying different RB subtypes remain poorly understood. The present study investigated the differential contribution of executive functions (EFs) to lower- and higher-level RBs in autistic children and explored whether specific EF domains mediated the relationship between sleep disturbances and RBs.

**Methods:**

Fifty-seven autistic children aged 6–14 years underwent neuropsychological and behavioral assessment. RBs were assessed using the Repetitive Behavior Scale–Revised, executive functions using the Behavior Rating Inventory of Executive Function–2, sleep disturbances through the Sleep Disturbance Scale for Children, and behavioral difficulties through the Child Behavior Checklist.

**Results:**

Partial correlations, regression analyses, and mediation models were performed. Distinct association patterns emerged across RB subtypes. Lower-level RBs were significantly associated only with emotional control difficulties, whereas higher-level RBs were associated with several EF domains, including inhibition, shifting, emotional control, planning, and initiation. Regression analyses showed that higher-level RBs were independently predicted by shifting difficulties, whereas lower-level RBs were significantly associated with IQ and behavioral problems. Mediation analyses indicated that emotional control difficulties significantly explained the association between sleep disturbances and lower-level RBs. In contrast, cognitive flexibility did not mediate the relationship between sleep disturbances and higher-level RBs.

**Conclusion:**

These findings provide preliminary evidence of different patterns of association across lower- and higher-level RBs in autistic children. Emotional regulation processes may be particularly relevant for lower-level RBs, whereas cognitive flexibility appears more strongly related to higher-level RBs. Findings also suggest that sleep disturbances may contribute to specific RB manifestations through different mechanisms.

## Introduction

Autism spectrum disorder (ASD) is a neurodevelopmental condition defined by the presence of two core features: persistent difficulties in social communication and social interaction across multiple contexts and by restricted, repetitive patterns of behavior, interests, or activities (American Psychiatric Association, 2022). Based on Turner’s conceptual framework (1999), repetitive behaviors (RBs) may be distinguished into two broad domains: lower-level RBs, characterized by repetitive sensorimotor actions such as motor stereotypies and recurrent object manipulation, and higher-level RBs, encompassing cognitive and behavioral rigidity, including rituals and insistence on sameness (Turner, 1999). RBs have been linked to a range of adverse developmental and psychosocial outcomes, including reduced learning experiences, heightened anxiety, obsessive-compulsive features, and increased caregiver burden and parental stress (Rodgers et al., 2012; Jiujias et al., 2017; Schulte et al., 2018; Baribeau et al., 2020; Scibelli et al., 2021; Chaxiong et al., 2022; Schwartzman et al., 2022).

Beyond core symptoms, ASD is characterized by the presence of frequent comorbidities (Fucà et al., 2023; Micai et al., 2023). Sleep disturbances are among the most common co-occurring conditions in autistic children (Lai et al., 2019; Micai et al., 2023), with prevalence estimated up to 80% (Johnson and Zarrinnegar, 2024). The types of sleep disorders predominantly seen in autistic children include insomnia, parasomnias, and circadian rhythm sleep–wake disorders (Sidhu et al., 2024). The mechanisms underlying sleep disturbances in ASD are complex and likely multifactorial. Proposed contributors include alterations in the homeostatic and circadian regulation of sleep, as well as biological factors such as circadian-related genetic variants, atypical melatonin and serotonin secretion, and differences in brain maturation. Together, these factors may increase vulnerability to persistent sleep difficulties in autistic children (Sidhu et al., 2024). Beyond their impact on nighttime sleep, sleep disturbances are recognized as an important determinant of daytime cognitive functioning. Experimental and clinical studies have consistently shown that insufficient or disrupted sleep negatively affects prefrontal-dependent processes, including inhibitory control, cognitive flexibility, working memory, and emotional regulation (Killgore, 2010). Although these associations have been documented in the general pediatric population (Mason et al., 2021), emerging evidence suggests that they may be particularly relevant in autistic children, in whom sleep problems and executive dysfunction frequently co-occur (Holingue et al., 2021). A growing body of evidence indicates that sleep disturbances are also associated with greater severity of RBs in autistic children. In particular, poorer sleep quality and more severe sleep disturbances have been linked to increased repetitive sensorimotor behaviors as well as behavioral rigidity and insistence on sameness, although the mechanisms underlying these associations remain poorly understood (Fucà et al., 2025; Passarini et al., 2025).

Taken together, these findings suggest that sleep disturbances may influence the expression of RBs through downstream effects on neurocognitive processes rather than through a direct pathway alone. In particular, executive dysfunction may constitute one neurocognitive pathway through which sleep disturbances associate with RBs. To better understand this potential mechanism, it is important to consider the role of executive functioning in the regulation of RBs. Executive functions (EFs) represent a set of higher-order cognitive processes that support goal-directed behavior, cognitive flexibility, self-regulation, and adaptive responses to environmental demands (Cristofori et al., 2019). EFs include the following processes: working memory (maintains and continuously updates information to support ongoing cognitive processes and goal-directed behavior), inhibitory control (suppression of irrelevant responses or information to enable adaptive, goal-directed behavior), cognitive flexibility (ability to adapt behavior and thinking in response to changing environmental demands, including shifting between tasks, strategies, or mental sets), planning (ability to formulate, evaluate, and organize goal-directed actions in order to achieve a desired outcome), reasoning (generalization and abstraction processes that enable concept formation and creativity), and problem solving (analyzing the elements of a problem and applying logical, mathematical, or systematic methods to derive a solution; Cristofori et al., 2019). EF deficits are consistently reported in autistic individuals, with evidence indicating a moderate and relatively uniform impairment across multiple executive domains when compared to neurotypical individuals (Demetriou et al., 2018). A substantial body of research has examined the association between RBs and EF, given that both elevated RBs and EF impairments are commonly observed in individuals with ASD and, to a lesser extent, in typical development. Across studies, poorer EF—particularly in domains such as cognitive flexibility (set shifting) and inhibitory control—has been consistently associated with higher levels of RBs. This relationship appears relatively stable across diagnostic groups and measurement approaches, suggesting a robust link between EF difficulties and the expression of RBs (Iversen and Lewis, 2021). However, some inconsistencies emerge between studies. For instance, while some studies reported that the association between EF difficulties and RBs remained significant after controlling for general cognitive ability, other findings suggest that the relationship may weaken when verbal intellectual abilities are taken into account. Moreover, most studies included only individuals in the average IQ range; finally few studies distinguish between lower- and higher-level RBs (Iversen and Lewis, 2021).

Although previous research has documented associations among sleep disturbances, EF impairments, and RBs in autistic children, some knowledge gaps remain. First, relatively few studies have examined whether specific EF domains are differentially associated with lower- and higher-level RBs, despite the heterogeneous nature of RBs. This distinction may be especially relevant given the heterogeneous nature of RBs and the possibility that different executive processes underlie repetitive sensorimotor behaviors versus cognitive and behavioral rigidity. Second, although sleep disturbances have been associated with both executive dysfunction and increased RB severity, no study, to our knowledge, has directly examined whether specific executive domains mediate the relationship between sleep disturbances and distinct RB subtypes.

Therefore, the present study aimed to investigate the differential contribution of executive functions to lower- and higher-level RBs in autistic children. Additionally, we examined whether specific EF domains mediated the relationship between sleep disturbances and RB subtypes. Based on previous literature, we hypothesized that EF difficulties would show distinct associations with lower- and higher-level RBs, and that executive dysfunction would mediate the relationship between sleep problems and RB severity.

## Materials and methods

### Procedure

Data were retrospectively collected through an in-depth review of a database including data from patients who referred to the Child and Adolescent Neuropsychiatry Unit of a tertiary-level children’s hospital between 2023 and 2025. These patients sought a neuropsychiatric evaluation following a pediatrician’s clinical suspicion of ASD or attended clinical follow-ups after receiving a diagnosis of ASD. Routine assessment procedure always included neuropsychiatric examination, cognitive and adaptive functioning evaluation, assessment of ASD symptoms and a psychopathological screening. All the questionnaires—administered in their validated Italian version—were completed by the primary caregiver. Inclusion criteria were as follows: ascertained diagnosis of ASD supported by gold-standard instruments; age comprised between 6 and 14 years. Exclusion criteria were as follows: presence of neurological conditions (e.g., epilepsy); presence of genetic syndromes. The study was conducted according to the guidelines of the Declaration of Helsinki and approved by the local Ethics Committee (protocol code: 2423_OPBG_2021, approved on 27 October 2021).

### Participants

The sample included 57 school-age autistic children (44 males and 13 females; mean age = 9.45, SD = 1.5). The mean IQ score of the participants was 83.49 (SD = 15.29), with 14% having an IQ < 70 and the remaining 86% having an IQ ≥ 70. The mean ADOS-2 Calibrated Severity Score was 6.3 (SD = 1.5).

### Measures

#### Baseline assessment: ASD diagnosis and cognitive abilities

The diagnosis of ASD was established in accordance with the DSM-5 TR (American Psychiatric Association, 2022) and was confirmed by the administration of the “gold-standard” instruments for the assessment of ASD symptoms, namely the Autism Diagnostic Observation Schedule, 2nd Edition-ADOS-2 (Lord et al., 2012) and the Autism Diagnostic Interview-Revised-ADI-R (Lord et al., 1994). The ADOS-2 is a semi-structured direct assessment of communication, social interaction, and play or imaginative use of materials for individuals with a suspected diagnosis of ASD. The ADOS-2 consists of five modules designed for children and adults with different levels of language, from nonverbal to verbally fluent; it was administered and scored by licensed clinicians. Total score combines symptoms from the Social Affect (SA) and Restricted and Repetitive Behaviors (RRB) domains. The instrument also generates Calibrated Severity Scores, which provide a relative metric of the severity of ASD-specific symptoms.

The ADI-R is a standardized, semi-structured interview during which caregivers report information about an individual suspected of having an ASD. The instrument generates algorithm scores for each of the three subdomains of autistic symptoms: qualitative impairments in reciprocal social behavior; qualitative abnormalities in communication and restricted range of interests and/or stereotypic behaviors. These topics address both current and previous functioning. The ADI-R can be used to assess children with a mental age of at least 18 months.

Cognitive development was assessed by the Leiter International Performance Scale-3rd Edition-Leiter-3 (Roid et al., 2013)—which provides a nonverbal measure of intelligence and assesses the ability to reason by analogy, by matching and perceptual reasoning in general, irrespective of language and formal schooling. The Global Non-Verbal Intelligent Quotient obtained through this test is based on four subtests: Figure Ground, Form Completion, Classification and Analogies, and Sequential Order.

#### Behavioral and psychological screening

RBs were assessed through the Repetitive Behavior Scale-Revised (RBS-R). The RBS-R (Bodfish et al., 2000; Fulceri et al., 2016) is a clinical rating scale including 43-items in a caregiver-completed questionnaire. Items are grouped into six subscales: Stereotyped Behavior (6 items—total score: 18), Self-injurious Behavior (8 items—total score: 24), Compulsive Behavior (8 items—total score: 24), Ritualistic Behavior (6 items—total score: 18), Sameness Behavior (11 items—total score: 33), and Restricted Behavior (4 items—total score: 12). Informants rate each behavior on a scale from 0 to 3, with ‘0’ indicating the behavior does not occur and ‘3’ indicating the behavior is a severe problem. A total score is calculated based on all items, ranging from 0 to 129. The internal consistency of the RBS-R total score provided good to excellent results with Cronbach’s alpha between 0.89 and 0.93 (Rojahn et al., 2013; Martínez-González and Piqueras, 2018). Internal consistency for subscale scores ranged between 0.55 and 0.88 (Rojahn et al., 2013; Inada et al., 2015). The questionnaire was filled by the main caregiver.

Behavioral and emotional difficulties were assessed through the Child Behavior Checklist – CBCL (Achenbach and Rescorla, 2007; D'Orlando et al., 2010). We used the CBCL 6–18, which is composed of eight Empirically Based Syndromes Scales (Anxious/Depressed, Withdrawn/Depressed, Somatic Complaints, Social Problems, Thought Problems, Attention Problems, Rule-Breaking Behavior and Aggressive Behavior), three general domains (Total, Internalizing and Externalizing problems), and six Diagnostic and Statistical Manual of Mental Disorders (DSM)-oriented scales (Affective Problems, Anxiety Problems, Somatic Problems, Attention Deficit/Hyperactivity Problems, Oppositional Defiant Problems and Conduct Problems). The CBCL uses T-scores to represent scales: the normal T-score range for all syndromes is 50–64, the borderline clinical range is 65–69, while the clinical range is 70–100. Internal consistency and test–retest reliability of the Empirically Based Syndromes Scales were found to be good, with Cronbach Alphas ranging from 0.78 to 0.94 (Achenbach et al., 2003). For the current study, we considered scores from the broadband scale “Total Problems.”

#### Evaluation of executive functions

The BRIEF-2 measures behavioral manifestations of EF impairments in youth ages 5–18 years (Gioia et al., 2015; Marano et al., 2016) using a 3-point Likert-scale measuring problem frequency: 1 = never; 2 = sometimes; 3 = often. The BRIEF-2 yields sex-adjusted and age-adjusted normative T scores (*M* = 50, SD = 10) for the nine scales (Inhibit, Self-Monitor, Shift, Emotional Control, Initiate, Working Memory, Plan/ Organize, Task Monitor, and Organization of Materials), three indexes (Behavior Regulation Index, BRI; Emotional Regulation Index, ERI; Cognitive Regulation Index, CRI) and the Global Executive Composite (GEC). Higher T scores denote greater executive function difficulties.

#### Screening for sleep disturbances

Sleep disturbances were assessed by means of the Sleep Disturbance Scale for Children-SDSC (Bruni et al., 1996), a parent-report questionnaire that has demonstrated through validation an adequate level of internal consistency, test–retest reliability, and availability of normative data. The SDSC explores the presence of sleep disorders during the previous 6 months and contains items with Likert scale values of 1–5. The questionnaire consists of 26 items subdivided into six sleep disorder subscales: disorders of initiating and maintaining sleep, disorders of arousal, sleep–wake transition disorders, disorders of excessive somnolence, and sleep hyperhidrosis. The sum of scores provides a total sleep score with a possible range from 26 to 130; a T-score of more than 70 is considered pathological. The SDSC is reported to have acceptable internal consistency for the total score, both with children with (alpha = 0.71) and without (alpha = 0.79) sleep disorders (Bruni et al., 1996).

### Statistical analyses

All analyses were performed using SPSS, version 22 (IBM Corp, 2021). Statistical significance was set at *p* < 0.05. Descriptive statistics were used to analyze demographic and clinical characteristics of the whole sample. Partial correlation analyses were conducted to examine the associations between EF domains and RBs while controlling for IQ. Linear regressions examined a model of child’s variables predicting both lower- and higher-level RBs; EF domains were selected according to the results of the correlation analyses. To test the hypothesized mediating model (see Figure 1), the PROCESS v4.2 macro, Model 4 (Hayes, 2013) for SPSS was used. We tested the indirect effect by generating the 95% confidence intervals using 5,000 bootstrap samples and estimated single-step indirect effects. Confidence intervals including zero indicated non-significant pathways (Field, 2013). To perform the hypothesized mediation models, we used EF measure as the mediator, sleep disturbances as the predictor, and RBs as the criterion. We employed a four-step regression approach to assess mediation: first, testing the total effect of sleep on RBs (path c); second, examining sleep’s effect on EFs (path a); third, evaluating EFs’ effect on RBs controlling for sleep (path b); and fourth, determining the direct effect of sleep on RBs while accounting for EFs (path c’).

**Figure 1:**
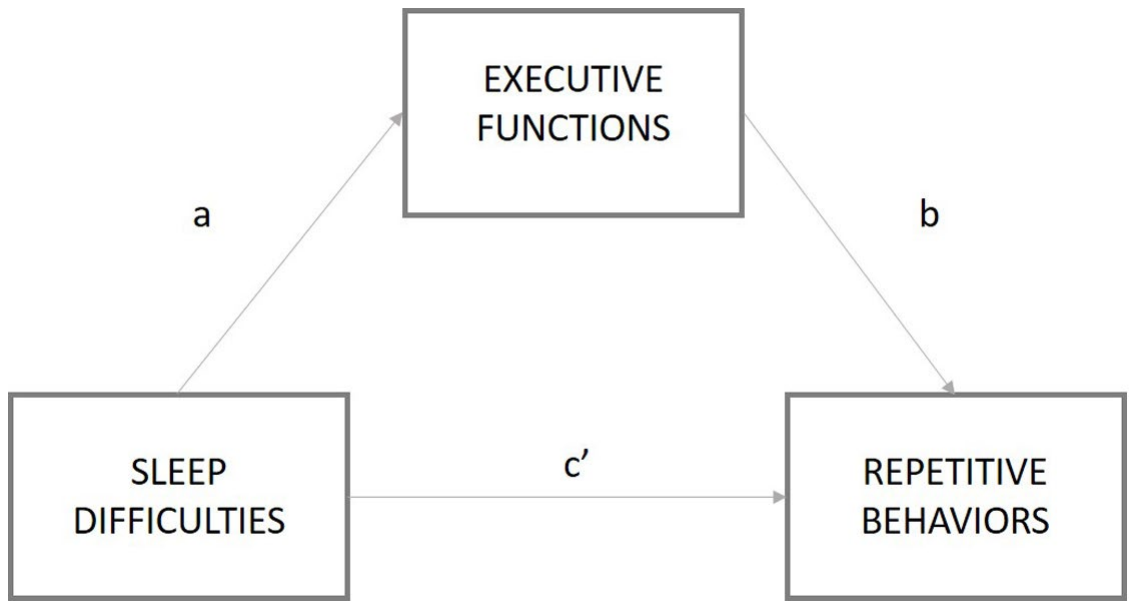
Hypothesized mediation model.

In reference to the RBs classification by Turner (1999) and Bodfish et al. (2000), the scores of the first two subscales of the RBS-R (i.e., Stereotyped and Self-injurious Behaviors) were summed to derive the score for the subscale associated with lower-order repetitive behaviors (L-RBS-R subscale). The remaining subscales (i.e., Compulsive, Ritualistic, Sameness and Restricted Behaviors) were summed to derive the score for the subscale associated with higher-order repetitive behaviors (H-RBS-R subscale) (Fucà et al., 2025).

## Results

### Association between repetitive behaviors and executive functions

Partial correlations controlling for IQ revealed different association patterns for low- and high-level RBs (see Table 1). Lower-level repetitive behaviors (L-RBS-R) were significantly associated only with emotional control difficulties. In contrast, higher-level repetitive behaviors (H-RBS-R) showed significant positive associations with several executive functioning domains, including inhibition, shifting, emotional control, planning, and task initiation.

**Table 1:** Partial correlations.

RBS	Inhibit *r* (*p*)	Self-monitor *r* (*p*)	Shift *r* (*p*)	Emotional control *r* (*p*)	Working Memory *r* (*p*)	Plan/organize *r* (*p*)	Initiate *r* (*p*)	Task monitor *r* (*p*)	Organization of materials *r* (*p*)
L-RBS-R	0.234 (0.086)	0.132 (0.336)	0.241 (0.077)	0.354 (0.008)	0.063 (0.649)	0.185 (0.176)	0.229 (0.092)	0.059 (0.671)	0.230 (0.091)
H-RBS-R	0.369 (0.006)	0.157 (0.252)	0.539 (<0.001)	0.473 (< 0.001)	0.099 (0.471)	0.390 (0.003)	0.363 (0.006)	0.162 (0.237)	0.106 (0.441)

L-RBS-R, lower-order repetitive behaviors; H-RBS-R, higher-order repetitive behaviors.

### Child’s features predicting repetitive behaviors

Regression models were informed by the results of the preliminary partial correlation analyses. Specifically, only EF domains significantly associated with RBs were included in the models, together with IQ, behavioral problems (i.e., CBCL Total Problems scale), and sleep difficulties (i.e., total score of the SDSC). For low-level repetitive behaviors (L-RBS-R), emotional control was entered as the EF predictor. The overall model was significant and explained 25.6% of the variance for lower-level RBs (adjusted *R*^2^ = 0.256, *p* = 0.001). IQ and total behavioral problems emerged as significant predictors, whereas emotional control did not significantly contribute to the model. For higher-level RBs, inhibition, shifting, emotional control, planning/organization, and initiation were included as predictors. The overall model significantly explained 30.1% of the variance in H-RBS-R scores (adjusted *R*^2^ = 0.301, *p* = 0.001). Among the executive functioning variables, only shifting emerged as a significant independent predictor of higher-level repetitive behaviors. Tables 2, 3 summarize the regression results. To facilitate direct comparison of the correlates of lower- and higher-level RBs, we re-ran the regression analyses using a common set of predictors comprising all variables included in either of the original regression models (IQ, CBCL Total Problems, SDSC Total Score, Inhibition, Shifting, Emotional Control, Initiation, and Planning/Organization). The same predictor set was therefore entered into both models. When the same set of predictors was entered into both models, shifting difficulties remained significantly associated with higher-level RBs, whereas no EF domain was significantly associated with lower-level RBs (see Supplementary Table S1).

**Table 2:** Regression analysis for the prediction of lower-level RBs.

Predictor	*B*	SE	*β*	*p*
IQ	−0.115	0.044	−0.307	0.012
Total behavioral problems (CBCL)	0.213	0.103	0.320	0.044
Sleep difficulties (SDSC)	−0.053	0.066	−0.111	0.418
Emotional control (BRIEF-2)	0.089	0.072	0.184	0.220

IQ, Intelligence quotient; CBCL, Child Behavior Checklist; SDSC, Sleep Disturbance Scale for Children; BRIEF-2, Behavior Rating Inventory of Executive Function, Second Edition.

**Table 3:** Regression analysis for the prediction of higher-level RBs.

Predictor	*B*	SE	*β*	*p*
IQ	−0.028	0.100	−0.033	0.781
Total behavioral problems (CBCL)	0.347	0.241	0.231	0.157
Sleep difficulties (SDSC)	0.160	0.147	0.147	0.284
Inhibition (BRIEF-2)	−0.220	0.247	−0.181	0.377
Shifting (BRIEF-2)	0.427	0.169	0.434	0.015
Emotional control (BRIEF-2)	0.221	0.237	0.201	0.355
Initiation (BRIEF-2)	−0.067	0.159	−0.066	0.676
Planning/organization (BRIEF-2)	−0.051	0.216	−0.043	0.813

IQ, Intelligence quotient; CBCL, Child Behavior Checklist; SDSC, Sleep Disturbance Scale for Children; BRIEF-2, Behavior Rating Inventory of Executive Function, Second Edition.

### The mediating role of executive functions

Two exploratory mediation models were conducted based on the results of the regression analyses, selecting EF dimensions differentially associated with lower- and higher-level RBs. A first mediation model included sleep disturbances (SDSC total score) as the predictor, lower-level RBs (L-RBS-R) as the outcome, and emotional regulation difficulties (BRIEF Emotional Control domain) as the mediator. As shown in Table 4, sleep disturbances significantly predicted emotional control difficulties (*B* = 0.42, SE = 0.12, *t* = 3.52, *p* = 0.001). Emotional regulation difficulties significantly predicted lower-level RBs (*B* = 0.19, SE = 0.07, *t* = 2.81, *p* = 0.007). The direct effect of sleep disturbances on L-RBS-R was not significant (*B* = −0.01, SE = 0.07, *t* = −0.08, *p* = 0.937). The overall model explained 14.86% of the variance in lower-level RBs. Importantly, the indirect effect of sleep disturbances on lower-level RBs through emotional control difficulties was significant (*β* = 0.08, 95% CI [0.0110, 0.1742]), suggesting that emotional control difficulties mediated the association between sleep disturbances and lower-level RBs.

**Table 4:** Mediation model examining the indirect effect of sleep disturbances on lower-level RBs through emotional control difficulties.

Path	*B*	SE	*t*	*p*
a: SDSC total → Emotional regulation	0.4238	0.1203	3.5225	0.0009
b: Emotional regulation → L-RBS-R	0.1902	0.0678	2.8063	0.0070
c′: SDSC total → L-RBS-R	−0.0054	0.0669	−0.0800	0.9365

SDSC, Sleep Disturbance Scale for Children; L-RBS-R, lower-order repetitive behaviors.

A second exploratory mediation model was conducted with sleep disturbances (SDSC total score) as the predictor, higher-level RBs (H-RBS-R) as the outcome, and Shift (BRIEF-2) as the mediator (see Table 5). The overall model explained 35.06% of the variance in higher-level RBs (*R*^2^ = 0.35). Although greater difficulties in cognitive flexibility were associated with higher levels of RBs, the indirect effect of sleep disturbances on higher-level RBs through Shift was not significant (*B* = 0.13, 95% CI [−0.0184, 0.2983]). Conversely, sleep disturbances retained a significant direct association with H-RBS-R, suggesting that cognitive flexibility difficulties did not account for the relationship between sleep problems and higher-order RBs.

**Table 5:** Mediation model examining the indirect effect of sleep disturbances on higher-level RBs through shift difficulties.

Path	*B*	SE	*t*	*p*
a: SDSC total → Shift	0.2673	0.1439	1.8574	0.0686
b: Shift → H-RBS-R	0.4841	0.1115	4.3432	0.0001
c′: SDSC total → H-RBS-R	0.2523	0.1226	2.0574	0.0445

SDSC, Sleep Disturbance Scale for Children; H-RBS-R, higher-order repetitive behaviors.

## Discussion

The present study aimed to preliminary explore the differential contribution of EFs to lower- and higher-level RBs in autistic children. Overall, findings revealed different patterns of association between EFs and RB domains. Specifically, emotional control difficulties were associated with lower-level RBs, whereas cognitive flexibility difficulties emerged as an important correlate of higher-level RBs. The association between lower-level RBs and emotional control difficulties may suggest that these behaviors are linked more closely to the regulation of internal states than to higher-order cognitive control processes. Previous behavioral literature has proposed that stereotyped behaviors may serve regulatory functions and can be maintained by different reinforcement contingencies, particularly automatic or sensory reinforcement mechanisms (Cunningham and Schreibman, 2008). From this perspective, repetitive sensorimotor behaviors may contribute to modulating emotional arousal or reducing discomfort, stress, or overstimulation. Consistent with this view, a review by Melo et al. (2020) highlighted that stereotyped movements in autistic individuals are often associated with emotional or environmental triggers, including anxiety, anger related to interpersonal events, and sensory stimulation. Such findings support the hypothesis that RBs may emerge, at least in some contexts, as responses to emotionally salient or dysregulating experiences. Therefore, children experiencing greater difficulties in emotional regulation may engage more frequently in lower-level RBs as a strategy for managing internal emotional or physiological states. However, emotional control did not retain an independent contribution in the regression model after accounting for broader child characteristics. Instead, lower-level RBs were significantly associated with IQ and behavioral difficulties, suggesting that these behaviors may emerge within a broader developmental and behavioral vulnerability profile. In line with this interpretation, Goldman et al. (2009) reported that lower cognitive functioning was independently associated with a greater frequency and variety of stereotyped behaviors in autistic children, supporting the idea that sensorimotor repetitive behaviors may be particularly influenced by general developmental functioning (Goldman et al., 2009). At the same time, stereotyped behaviors are highly heterogeneous in their functional properties, and similar behavioral topographies may reflect different underlying mechanisms across individuals and contexts (Cunningham and Schreibman, 2008).

On the other hand, we found that higher-level RBs were primarily linked to cognitive flexibility difficulties. This finding is theoretically coherent with the nature of higher-level RBs, which include insistence on sameness, ritualistic behaviors, compulsions, and restricted interests. Unlike lower-level RBs, which are often conceptualized as sensorimotor or self-regulatory responses, higher-level RBs appear to reflect difficulties adapting thoughts and behaviors to changing environmental demands (Turner, 1999; Leekam et al., 2011). Our findings are consistent with recent evidence highlighting cognitive flexibility as a key correlate of higher-order RBs in ASD. For example, Saniee et al. (2026) examined cognitive flexibility in young autistic children using both a performance-based measure (i.e., the Dimensional Change Card Sort task) and a parent-report assessment, allowing the authors to capture cognitive flexibility across laboratory and everyday contexts (Saniee et al., 2026). Their results showed that deficits in cognitive flexibility were specifically associated with higher-order RBs, including ritualistic routines and insistence on sameness, whereas lower-order sensorimotor RBs showed no significant associations. Notably, higher-order RBs remained among the strongest contributors to cognitive flexibility difficulties even when other executive domains and ASD-related characteristics were considered. The convergence between performance-based and parent-reported measures further suggested that the relationship between difficulties in cognitive flexibility and higher-order RBs is robust across assessment methods. Together with the present findings, these results support the hypothesis that cognitive flexibility difficulties may represent a core correlate of higher-level RBs in ASD. Of note, although several EF domains showed significant bivariate associations with higher-level RBs, shifting difficulties remained significantly associated with higher-level RBs in the multivariate model. Importantly, when the same set of predictors was entered into both lower- and higher-level RB models, shifting difficulties remained significantly associated with higher-level RBs, whereas no EF domain was significantly associated with lower-level RBs. This pattern suggests that difficulties in shifting may be particularly relevant to higher-level RBs, although the present findings do not establish a specific or unique predictive role of this EF domain. However, it is important to note that some conceptual overlap exists between behavioral manifestations of shifting difficulties assessed by the BRIEF-2 and behaviors captured by the RBS-R Immutability/Sameness domains, particularly with respect to difficulty transitioning between activities and resistance to changes in routines.

The second aim of this study was to explore whether specific EF domains mediated the relationship between sleep disturbances and RB subtypes. Our findings, albeit preliminary, suggest different patterns of associations linking sleep difficulties to lower- and higher-level RBs. Specifically, emotional control mediated the association between sleep disturbances and lower-level RBs, whereas cognitive flexibility did not mediate the relationship between sleep disturbances and higher-level RBs. The mediation effect observed for lower-level RBs suggests that sleep disturbances may influence repetitive sensorimotor behaviors indirectly through difficulties in emotional regulation. Previous research has consistently shown that poor sleep quality and sleep disruptions negatively affect emotional and behavioral regulation in autistic children, increasing irritability, emotional lability, and difficulties managing internal states (Holingue et al., 2021; Johnson and Zarrinnegar, 2024). More specifically, sleep problems in ASD have been associated with broader patterns of daytime behavioral dysregulation. For instance, Mazurek and Sohl (2016) reported significant associations between sleep disturbances and behavioral difficulties including irritability, aggression, hyperactivity, and inattention, with night awakenings showing particularly strong relationships with daytime problems (Mazurek and Sohl, 2016). Similarly, Cohen et al. (2018), using longitudinal sleep recordings across more than 20,000 nights in individuals with low-functioning ASD, observed that sleep patterns significantly predicted the occurrence of challenging behaviors in the majority of participants (Cohen et al., 2018). Together, these findings suggest that sleep disruptions may alter the child’s capacity to regulate emotional and behavioral responses during the day. In this context, lower-level RBs may represent behavioral responses that contribute to regulating heightened emotional or physiological arousal. This interpretation aligns with previous conceptualizations of stereotyped behaviors as potentially serving self-regulatory functions (Cunningham and Schreibman, 2008) and with evidence suggesting that emotional and environmental factors frequently trigger repetitive sensorimotor behaviors (Melo et al., 2020). Thus, sleep difficulties may increase vulnerability to emotional dysregulation, which in turn may contribute to the expression of lower-level RBs.

Although cognitive flexibility emerged as the strongest executive predictor of higher-level RBs, it did not explain the association between sleep disturbances and these behaviors, suggesting that cognitive flexibility alone does not account for the pathway linking sleep difficulties to higher-order RB manifestations. This finding may reflect the multifactorial nature of higher-level RBs, such as rituals, insistence on sameness, and compulsive behaviors, which likely involve broader cognitive and emotional processes beyond executive functioning alone. Previous literature has suggested that higher-level RBs may be associated not only with cognitive rigidity but also with increased anxiety, intolerance of uncertainty, and a heightened need for predictability (Rodgers et al., 2012; Wigham et al., 2015). Consistent with this interpretation, Sevincok et al. (2026) reported significant associations between specific sleep disturbances and higher-level RBs in autistic children and adolescents (Sevincok et al., 2026). In particular, sameness behaviors were associated with both sleep timing difficulties and overall sleep problems, whereas compulsive behaviors were linked to arousal and waking disturbances. These findings suggest that sleep difficulties may be particularly relevant for RBs characterized by rigidity and insistence on predictability, supporting the hypothesis that sleep disturbances may contribute to higher-level RBs through mechanisms that extend beyond cognitive flexibility alone. Furthermore, Richdale et al. (2023) emphasized that sleep disturbances in ASD are closely embedded within broader affective and regulatory difficulties, including heightened anxiety, emotional dysregulation, and increased physiological arousal, highlighting that sleep problems should not be conceptualized as isolated symptoms but rather as part of a complex, multidimensional regulatory system (Richdale et al., 2023). In this framework, sleep disturbances may contribute to increased stress vulnerability and reduced adaptive coping capacities, thereby strengthening the child’s tendency to rely on rigid routines and repetitive behavioral patterns as compensatory strategies. Therefore, poor sleep may increase the expression of higher-level RBs through affective and behavioral mechanisms that are only partially captured by EF measures.

Taken together, these findings suggest that sleep disturbances may not affect all kinds of RBs through a common pathway. Rather, lower-level and higher-level RBs may involve partially distinct mechanisms, with emotional regulation appearing more relevant for repetitive sensorimotor behaviors and broader behavioral or affective processes potentially contributing to higher-level manifestations, such as the insistence on sameness.

Some limitations should be acknowledged when interpreting the results of this study. First, the small sample size may have limited the generalizability of the findings, particularly with respect to the mediation analyses and the examination of multiple EF domains. Second, the cross-sectional design precludes conclusions regarding the causal nature of the observed associations among sleep disturbances, EF, and RBs. Longitudinal studies are needed to clarify potential developmental pathways underlying these relationships. Third, the study relied primarily on parent-report measures: although these instruments provide clinically meaningful information regarding children’s functioning in everyday contexts, shared method variance may have contributed to the observed associations. However, recent research suggested strong convergence between performance-based and parent-reported assessment of cognitive flexibility (Saniee et al., 2026). Future studies could integrate performance-based assessments of EFs and objective sleep measures, such as actigraphy or polysomnography. A further limitation concerns the restricted age range of the sample, which included only school-age children (6–14 years). As a result, preschool-aged children were not represented, limiting the generalizability of the findings across developmental stages. This is relevant given that RBs and their underlying cognitive and emotional correlates may vary across development, particularly in early childhood. Moreover, some conceptual overlap between behavioral manifestations of shifting difficulties assessed by the BRIEF-2 and behaviors captured by the RBS-R, particularly those related to resistance to change and difficulty transitioning between activities, cannot be excluded. Therefore, the observed associations should not be interpreted as demonstrating uniquely specific relationships between EF domains and RBs. Despite these limitations, the present study also has several strengths. First, unlike many previous studies investigating RBs as a unitary construct, the present work distinguished between lower- and higher-level RBs, allowing a more nuanced examination of their differential associations with EF. Categorizing RBs into broad domains may fail to capture their inherent heterogeneity, as important distinctions between behaviors within the same category can be overlooked (Jasim and Perry, 2023). Second, the study adopted a multidimensional approach by simultaneously considering executive functions, sleep disturbances, behavioral difficulties, and cognitive functioning, thus providing a broader characterization of factors potentially associated with RBs in autistic children. Additionally, the inclusion of children with a relatively wide range of cognitive abilities, including participants with intellectual disability, may enhance the ecological validity of the findings and increase their relevance for clinically heterogeneous autistic populations.

The present findings have relevant clinical implications. The differential associations observed across RB subtypes suggest that lower- and higher-level RBs may rely on partially distinct mechanisms and therefore could benefit from different intervention targets. Specifically, interventions aimed at improving emotional regulation skills may indirectly reduce lower-level RBs, whereas approaches targeting cognitive flexibility may be particularly relevant for higher-level RBs characterized by poor flexibility and insistence on sameness. Furthermore, given the observed associations between sleep disturbances and RBs, sleep difficulties should be routinely assessed within clinical evaluations of autistic children, as improving sleep quality may have broader effects on behavioral and executive functioning outcomes. Future research should replicate these findings in larger samples and employ longitudinal designs to better clarify the temporal and potentially reciprocal relationships among sleep disturbances, executive functioning, and RBs.

## Data Availability

The raw data supporting the conclusions of this article will be made available by the authors upon reasonable request.
