# Supplementary material for: Sleep, executive functions, and repetitive behaviors in autism spectrum disorder: different patterns of association with lower- and higher-level repetitive behaviors
Source: Front Psychol. 2026 Oct 7;17:1903369. doi: 10.3389/fpsyg.2026.1903369 (PMC13683773; doi:10.3389/fpsyg.2026.1903369)
Supplement: Supplementary file 1 [file Table_1.docx]

Supplementary Material

# 1 Supplementary Table S1

*Multiple regression analyses using a common set of predictors for lower- and higher-level repetitive behaviors*

|  | **L-RBS-R** |  |  | | **H-RBS-R** |  |  |  |
| --- | --- | --- | --- | --- | --- | --- | --- | --- |
| Predictor | B | SE | β | p | B | SE | β | p |
| IQ | −0.110 | 0.047 | −0.294 | **.023** | −0.028 | 0.100 | −0.033 | .781 |
| Total behavioral problems (CBCL) | 0.215 | 0.113 | 0.323 | .063 | 0.347 | 0.241 | 0.231 | .157 |
| Sleep difficulties (SDSC) | −0.041 | 0.069 | −0.084 | .560 | 0.160 | 0.147 | 0.147 | .284 |
| Inhibition (BRIEF-2) | −0.070 | 0.116 | −0.129 | .551 | −0.220 | 0.247 | −0.181 | .377 |
| Shifting (BRIEF-2) | −0.007 | 0.079 | −0.016 | .932 | 0.427 | 0.169 | **0.434** | **.015** |
| Emotional control (BRIEF-2) | 0.175 | 0.111 | 0.359 | .122 | 0.221 | 0.237 | 0.201 | .355 |
| Initiation (BRIEF-2) | 0.045 | 0.075 | 0.100 | .549 | −0.067 | 0.159 | −0.066 | .676 |
| Planning/Organization (BRIEF-2) | −0.089 | 0.101 | −0.167 | .385 | −0.051 | 0.216 | −0.043 | .813 |
| **R²** | .330 |  |  |  | .401 |  |  |  |
| **Adjusted R²** | .218 |  |  |  | .301 |  |  |  |
| **F** | 2.949 |  |  |  | 4.014 |  |  |  |
| ***p* (model)** | .009 |  |  | | .001 |  |  |  |

*Note*. IQ = Intelligence quotient; CBCL = Child Behavior Checklist; SDSC = Sleep Disturbance Scale for Children; BRIEF-2 = Behavior Rating Inventory of Executive Function, Second Edition
